# Design of Electronic Nose Detection System for Apple Quality Grading Based on Computational Fluid Dynamics Simulation and K-Nearest Neighbor Support Vector Machine

**DOI:** 10.3390/s22082997

**Published:** 2022-04-14

**Authors:** Xiuguo Zou, Chenyang Wang, Manman Luo, Qiaomu Ren, Yingying Liu, Shikai Zhang, Yungang Bai, Jiawei Meng, Wentian Zhang, Steven W. Su

**Affiliations:** 1 College of Artificial Intelligence, Nanjing Agricultural University, Nanjing 210031, China; 2020812083@stu.njau.edu.cn (C.W.); 32316118@njau.edu.cn (M.L.); 32315407@njau.edu.cn (Q.R.); lyy@njau.edu.cn (Y.L.); 2018812099@stu.njau.edu.cn (S.Z.); 2 College of Engineering, Nanjing Agricultural University, Nanjing 210031, China; 2020112043@stu.njau.edu.cn; 3 Department of Mechanical Engineering, University College London, London WC1E 7JE, UK; jiawei.meng@ucl.ac.uk; 4 Faculty of Engineering and Information Technology, University of Technology Sydney, Sydney, NSW 2007, Australia; wentian.zhang@alumni.uts.edu.au

**Keywords:** apple quality grading, electronic nose, computational fluid dynamics, K-nearest neighbor, support vector machine

## Abstract

Apples are one of the most widely planted fruits in the world, with an extremely high annual production. Several issues should be addressed to avoid the damaging of samples during the quality grading process of apples (e.g., the long detection period and the inability to detect the internal quality of apples). In this study, an electronic nose (e-nose) detection system for apple quality grading based on the K-nearest neighbor support vector machine (KNN-SVM) was designed, and the nasal cavity structure of the e-nose was optimized by computational fluid dynamics (CFD) simulation. A KNN-SVM classifier was also proposed to overcome the shortcomings of the traditional SVMs. The performance of the developed device was experimentally verified in the following steps. The apples were divided into three groups according to their external and internal quality. The e-nose data were pre-processed before features extraction, and then Principal Component Analysis (PCA) and Linear Discriminant Analysis (LDA) were used to reduce the dimension of the datasets. The recognition accuracy of the PCA–KNN-SVM classifier was 96.45%, and the LDA–KNN-SVM classifier achieved 97.78%. Compared with other commonly used classifiers, (traditional KNN, SVM, Decision Tree, and Random Forest), KNN-SVM is more efficient in terms of training time and accuracy of classification. Generally, the apple grading system can be used to evaluate the quality of apples during storage.

## 1. Introduction

A considerable number of countries in the world regard apples as a major consumer fruit because of their strong ecological adaptability, high nutritional value, good storability, and long supply cycle [1]. In terms of the fruit industry, the study focusing on the detection of apple quality is crucial and urgent. The chemical analysis method is used because the traditional fruit quality assessment method has shortcomings of sample damage, complicated operation procedures, long testing periods, and the inability to achieve real-time detection. Among non-destructive testing methods, physical detection methods (e.g., near-infrared spectroscopy and hyperspectral imaging) have emerged and rapidly developed in recent years and have become popular in this field [2,3,4,5]. Nevertheless, the information obtained by near-infrared spectroscopy and hyperspectral spectroscopy is easily obscured by the spectral changes caused by the physical properties of food [6]. Furthermore, most of the instruments used in this method are complicated and expensive.

The electronic nose (e-nose), a novel intelligent bionic technology, has developed rapidly in recent years. It detects simple and complex odors by a selective gas sensor array and the associated pattern recognition algorithms. It has the advantages of simple and destructive operation procedures, fast detection speeds, and good recognition effects [7,8]. The composition and concentration of aromatics volatilized by apples are closely related to the variety, quality, and maturity of the apples, and many researchers have applied the e-nose to the classification of different fruits, ripeness testing, and quality testing of fruits [9]. Baietto et al. [10] explored the current and potential utilization of e-nose devices with specialized sensor arrays as new effective tools for more efficient fruit aroma analysis to replace conventional expensive methods used in fruit aroma assessments. Solis-Solis et al. [11] used an e-nose to identify eight varieties of apricots by using PCA and factor analysis. Calu et al. [12] used an e-nose with 18 metal oxide gas sensors to test the apples of seven different varieties from different regions. The obtained data were analyzed by Principal Component Analysis (PCA) and Discriminant Factor Analysis (DFA), and the e-nose showed the ability to distinguish apple varieties. Hui et al. [13] used a self-made e-nose to detect the ripeness of apples, and the results showed that the analysis of the data using the stochastic resonance signal-to-noise spectrum could accurately distinguish between fresh apples, partially ripe apples, and overripe apples. Sanaeifar et al. [14] applied an e-nose system to identify bananas with different ripeness degrees. Through the analysis and comparison using a Support Vector Machine (SVM), the classification accuracy of each type of sample was 98.66%. Fu et al. [15] applied a self-made e-nose system to the prediction and evaluation of the freshness of grapes, and the accuracy of recognition reached 83.3% using PCA and multiple discriminant analysis methods. Shao et al. [16] used a self-made e-nose to measure the quality of mangoes and used PCA and stochastic resonance for data analysis. The linear fitting SR feature was used to establish a mango quality prediction model, and the accuracy of identification reached 90%. Jia et al. [17] used the PEN3 e-nose to detect and recognize fresh and moldy apples with a maximum prediction accuracy of 96.3%.

In summary, the e-nose can be used for the quality detection, grading, and classification of fruits. However, the existing research is limited to sensors, recognition algorithms, and applications [18]. Additionally, attention is rarely paid to the nasal cavity structure, which also has a significant influence on the performance of e-noses. Keyhani et al. [19] showed that the process of organisms producing olfaction depended on the nasal cavity structure, the velocity field of the gas flow, the diffusivity and adsorption of odor molecules in the air and mucosa, and the thickness of the tissues. Similarly, the olfactory ability of an e-nose is also related to its nasal cavity structure and airflow velocity field. Consequently, the influence of the nasal cavity structure on the olfactory ability of an e-nose should be considered when designing an e-nose system. Recently, there have been some studies on this issue. Wu et al. [20] used CFD to optimize the sensing chamber in the e-nose, ensuring the uniformity of airflow distribution in the chamber structure where the gas sensor array was embedded. Dohare et al. [21] designed different sensing chambers, and the effect of the baffle positions in the flow distribution was investigated through numerical simulations. Annanouch et al. [22] applied a new 3D-printed prototype chamber (boat-shaped design) compared with the commonly used testing chamber (cross-shape design), leading to an increase in the dynamics. The performance of the whole system was improved. A novel e-nose system was developed in this study. The fluid characteristics of the nasal cavity structure in the e-nose system were numerically simulated. Then, the KNN-SVM (K-nearest neighbor SVM) classifier was proposed to overcome the shortcomings of the traditional SVMs. This provides a new technique for improving the efficiency of apple quality grading in the harvesting and storage process. The main contributions of this study are as follows:

(1) An e-nose system was designed for apple quality grading, with the merits of convenient sample treatment, low cost, and a good recognition effect.
(2) Two different nasal cavity structures of the e-nose were designed, and the optimal one was selected for apple grading on the basis of computational fluid dynamics (CFD).
(3) The features of the 18-dimensional gas data were extracted from the data collected by the designed e-nose, and then the KNN-SVM classifier was proposed in this study to achieve accurate and non-destructive apple grading.

## 2. Materials and Methods

### 2.1. Materials

Red Fuji apples of different qualities were used as the research objects. The fruits were graded by human experts on the basis of their external quality and internal quality into three grades, i.e., L1, L2, and L3, as detailed in Table 1. The volatilized gases of apples mainly include CO_2_, alcohols, hydrocarbons, lipids, and a small number of amines and nitrogen gases. The odor of an apple is closely related to its quality [23,24]; when apples are degraded (e.g., rotted or broken), there is a change in the concentration of the gases they emit, such as ethylene.

### 2.2. Design of the Device

#### 2.2.1. Overall Design of the Device

**Figure 1:**
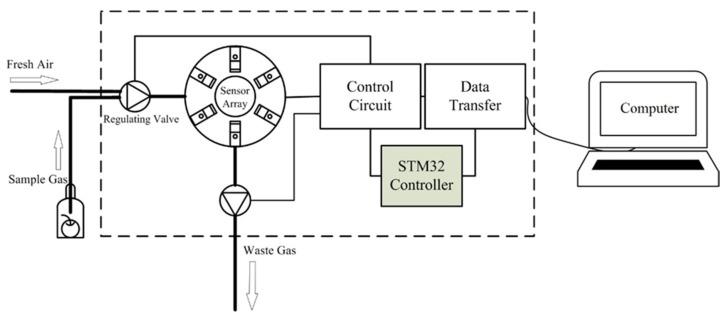
The schematic illustration of the developed electronic nose system.

Figure 1 is the schematic illustration of the developed e-nose system. The system consists of two subsystems: the hardware that reacts with gases and the software that analyzes the data. The hardware includes a gas cylinder, a gas pipe, regulating valves, a sensor array, control circuits, an A/D acquisition card, and a computer. The software was developed to pre-process the data obtained by the sensor array and extract the features from these datasets. The KNN-SVM method was used to grade the quality of apples.

#### 2.2.2. Nasal Cavity Structure

**Figure 2:**
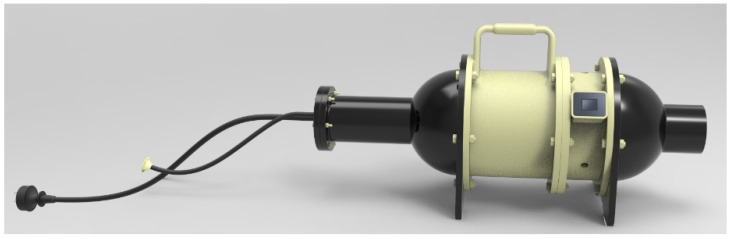
The structure of the nasal cavity with the gas inlet on the left and the exhaust outlet on the right.

The device was designed as a highly-symmetrical circular tube to guarantee a uniform and stable gas flow inside of the device [24]. The structure of the nasal cavity is detailed in Figure 2, where the left part is the gas inlet and the right part is the exhaust outlet.

##### Gas Sensor Array

The core component of the sensor unit is an array of sensitive devices consisting of six SnO_2_ gas sensors (MQ-9, MQ-3, MQ-6, MQ-8, MQ-2, and MQ-135). According to the gases emitted by the apples, six gas sensors were selected to form a circular sensor array for detecting the concentrations of CO, CO_2_, alcohols, hydrocarbons, harmful gases (including ammonia, sulfides, benzene vapors, etc.), and hydrogen gas. The details of the sensor are shown in Table 2.

**Figure 3:**
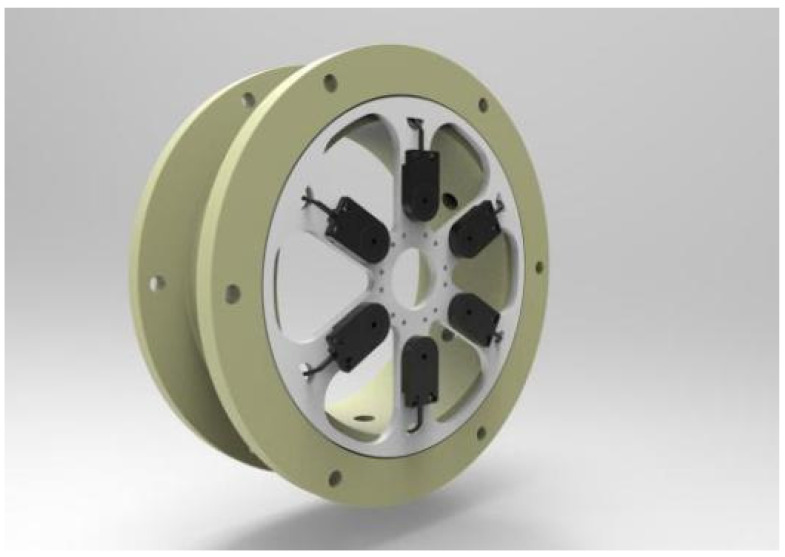
The specific structure of the sensor array with six SnO_2_ gas sensors.

The six sensors were evenly mounted on the ring to reduce the error caused by the sensor position. The specific structure of the sensor array is detailed in Figure 3.

##### Steady Flow Plate

**Figure 4:**
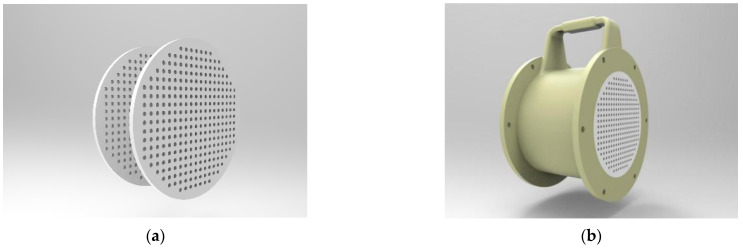
The structure of the porous steady flow plates. (**a**) Two layers of the steady flow plates, (**b**) the installation of the steady flow plates.

The SnO_2_ sensor detects the gas through the change in the conductivity caused by the adsorption and desorption of gas molecules on the semiconductor surface [25]. The flow condition in the sensing chamber strongly influences the e-nose’s ability to recognize and quantify odors [26]. Therefore, the detection accuracy of the sensor is related to the stability of the gas flow. Porous steady flow plates were designed in this study, as shown in Figure 4. The two kinds of nasal cavity structures with and without steady flow plates were compared and simulated by CFD simulation to select the optimal one.

##### Air Inlet Dimensions

**Figure 5:**
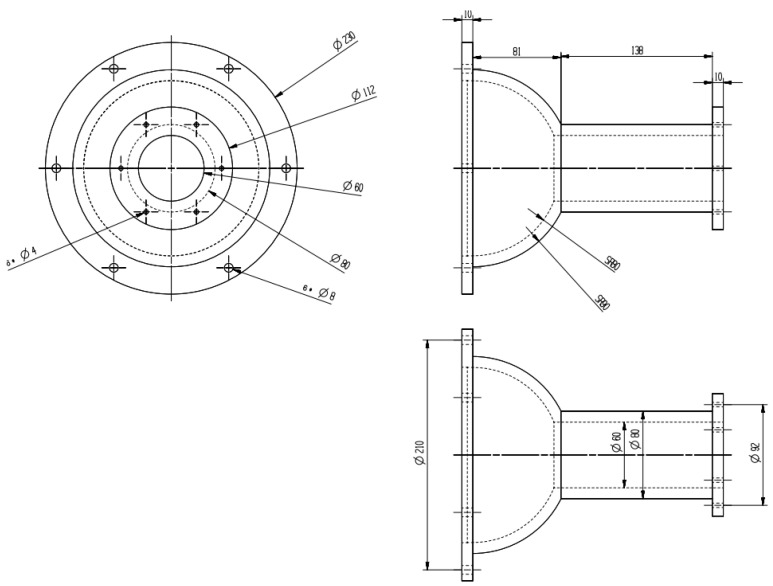
Dimensional design of the air inlet.

The size of the inlet is required for the calculation of the Reynolds number for flow field simulation; hence, we demonstrate the size of the inlet in Figure 5. According to the size and other characteristics of this device, it is appropriate to use laminar flow for simulation.

#### 2.2.3. Experimental Verification by CFD Simulation

CFD is a discipline that uses numerical methods to solve governing equations to discover the laws of various flow phenomena. The designed e-nose system was analyzed by CFD simulation to explore whether the addition of the steady flow plates could improve the detection accuracy of the e-nose. ANSYS 2019 R1 software was used for grid division, model solving, and post-processing for CFD simulation in this study with an Inter (R) Core (TM) i5-6500 CPU @ 3.20 GHz × 4, 16 G memory, NVIDIA GeForce GTX 750 Ti graphics card.

##### Construction of the 3D Electronic Nose Model

**Figure 6:**
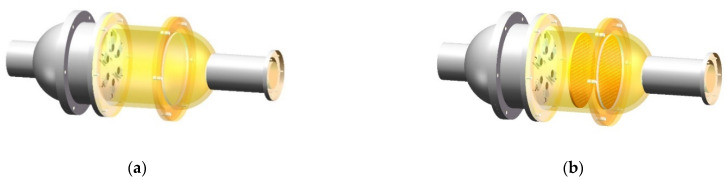
3D electronic nose model. (**a**) The model without steady flow plates; (**b**) the model with steady flow plates.

The 3D e-nose model was designed using SolidWorks according to the above analysis, as demonstrated in Figure 6, where (a) is a model without steady flow plates, and (b) is a model with steady flow plates.

##### Governing Equations

The governing equations of gas molecule transmission include continuity Equation (1), mass conservation Equation (2), and component mass conservation Equation (3) [27], as expressed by:

(1) $$\frac{\partial u_{i}^{*}}{\partial x_{i}^{*}}=0$$

(2) $$\frac{\partial \left(\rho u\right)}{\partial x}+\frac{\partial \left(\rho v\right)}{\partial y}+\frac{\partial \left(\rho w\right)}{\partial z}=0$$

(3) $$\frac{\partial \left(\rho c_{s}\right)}{\partial t}+div\left(pu_{i}c_{s}\right)=div\left(D_{s}grad\left(\rho c_{s}\right)\right)+S_{s}$$

where $x_{i}$ is the position vector, $u_{i}$ is the velocity vector, ρ is the fluid density, t is the time, *u*, *v*, and *w* are the components of the velocity vector in the *x*, *y*, and *z* directions, respectively, $c_{s}$ is the volume concentration of component *s*, $\rho c_{s}$ is the mass concentration of the component, $D_{s}$ is the diffusion coefficient of the component, and $S_{s}$ is the mass of the component produced by the chemical reaction per unit volume in unit time. The Reynolds number is $Re=\frac{\rho u_{in}D_{in}}{\mu }$, where $u_{in}$ is the average velocity at the inlet, $D_{in}$ is the inlet diameter, and μ is the dynamic viscosity.

##### Meshing

**Figure 7:**
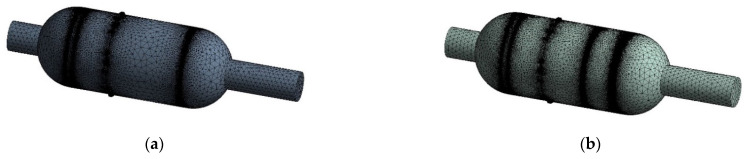
Meshing for two models. (**a**) Meshing without steady flow plates, (**b**) meshing with steady flow plates.

Meshing was performed using the ANSYS workbench mesh. The physics preference was divided by CFD. Because of the complex internal structure, FILL was used to obtain the internal watershed in the DM, and non-structural meshing was adopted for the watershed. The model without steady flow plates was divided into 813,176 nodes and 4,271,631 cells, as shown in Figure 7a. The model with steady flow plates was divided into 1,907,501 nodes and 9,878,409 cells, as shown in Figure 7b.

##### Setting Boundary Conditions

The parser uses Fluent in the ANSYS component. The Reynolds number obtained in the calculation of the e-nose model was less than 2320, so the numerical simulation process was a laminar flow process. The boundary conditions for simulating the flow field are shown in Table 3.

### 2.3. Data Analysis

#### 2.3.1. Data Collection and Feature Extraction

**Figure 8:**
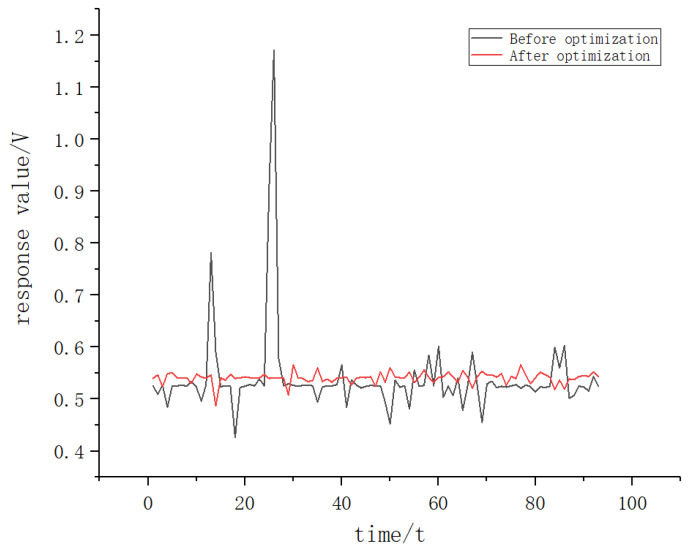
Comparison of data collection before and after optimization design.

Before the formal test, MQ-135 was used to conduct a pre-collection test to compare the data collection before and after the optimized design of the e-nose. As can be seen in Figure 8, the data collected by the optimized device were more stable than those before optimization, which is conducive to feature extraction later.

**Figure 9:**
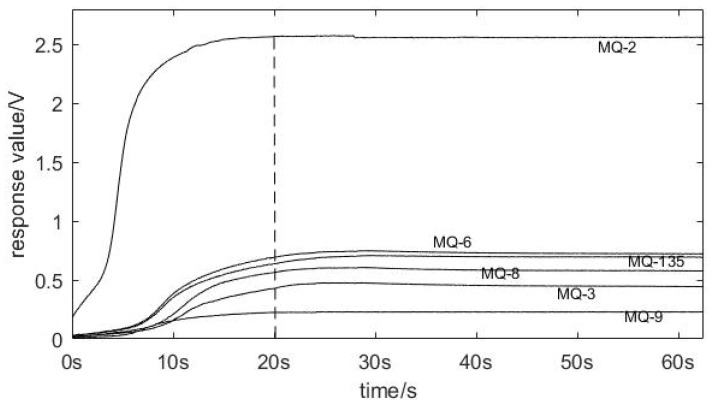
The sensor response values of L1 apple quality with time.

**Figure 10:**
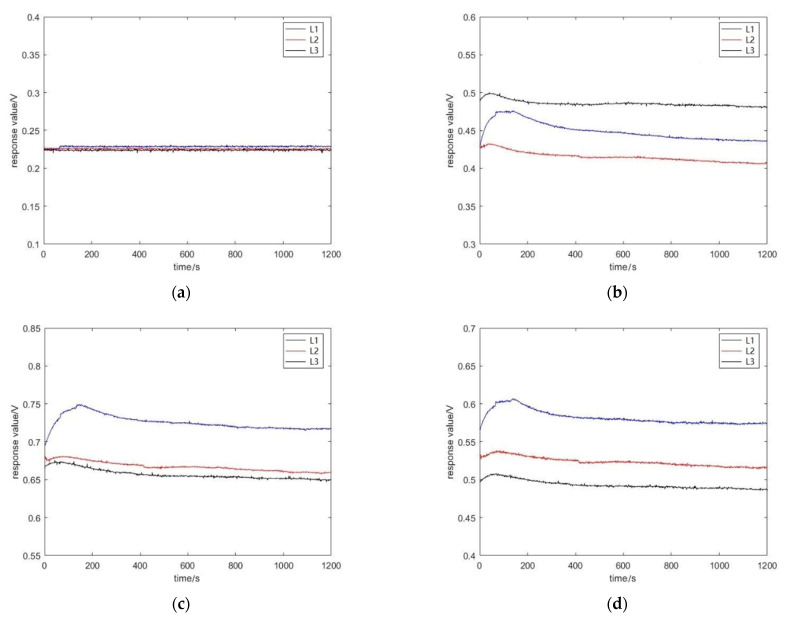

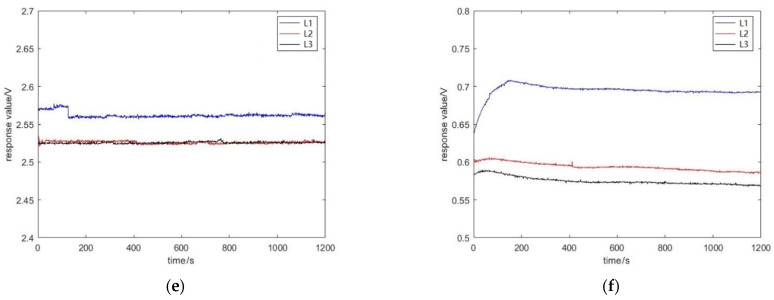
The raw response curves of six sensors for L1, L2, and L3. (**a**) MQ-9, (**b**) MQ-3, (**c**) MQ-6, (**d**) MQ-8, (**e**) MQ-2, (**f**) MQ-135.

The odor information of the three groups of apples was collected using the designed device. Firstly, the gas in the sample box was exhausted. After the apples were placed in the sample box for 2 min, the device began to extract the gas in the sample box, and the gas passed through the steady flow plates then contacted the sensor array, causing a change in the output voltage of the sensor, and the output voltage ranged from 0 V to 3.3 V. The output voltage was then transferred to the computer after AD conversion. Take the change in the sensor data for L1 apple quality over time as an example. Figure 9 shows that the data were gradually stable after 20 s. The data were selected as the response data of the sensor after 20 s to facilitate the extraction of data features. Figure 10 demonstrates the raw response curves of six sensors for L1, L2, and L3. The ordinate is the voltage value output of the sensor, and the abscissa is the frequency of collection.

**Figure 11:**
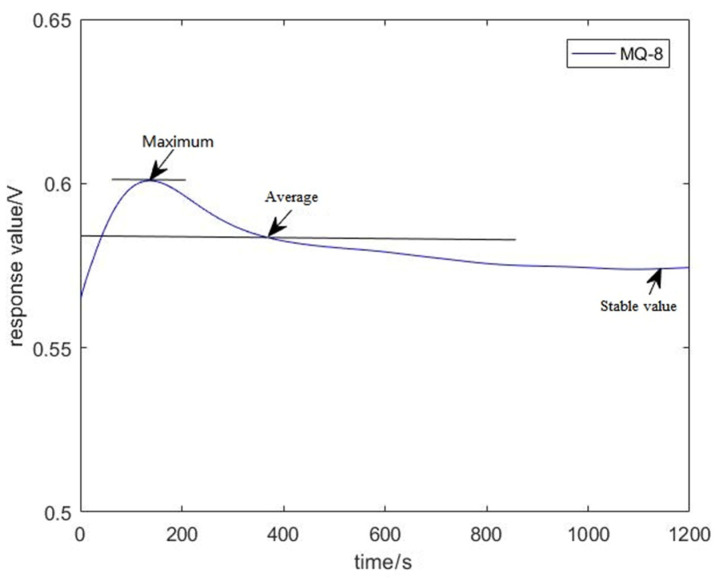
The features of Sensor MQ-8 extracted from the pre-processed curve.

**Figure 12:**
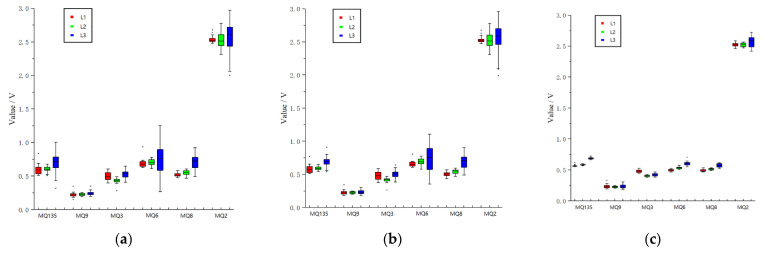
Boxplot showing the feature values of six sensors. (**a**) Maximum, (**b**) Average, (**c**) Stable value.

The features were extracted from the original curves to verify whether the device could grade the quality of apples. Jitter was observed in the sampled value because of the existence of interference, as indicated in Figure 10. The SG (Savitzky–Golay) filtering algorithm [28] was used to obtain the final data. The SG algorithm uses the least squares method and moves the window on the basis of a polynomial in the time domain. This method preserves the detailed features of signals while filtering out the noise. Figure 11 is the pre-processed curve. After repeated comparisons, the maximum value, the stable value, and the average value were extracted from the curve as a set of features. Thus, the 18-dimensional features were obtained by the six sensors. For each apple, ten features were collected, and a total of 300 sets of the feature were collected. In order to observe the relationship between feature parameters of each sensor more intuitively, the feature values were visualized, as shown in Figure 12.

#### 2.3.2. Principal Component Analysis (PCA)

PCA transforms multidimensional indicators into a few comprehensive indicators [29]. Since gas sensors often have cross-sensitivity with each other [30], the sole analysis at the variable level ignores the potential link between variables. When using PCA to analyze the data, the raw data can be transformed into a set of linearly independent dimensions. The method for extracting PCA feature vectors is described as follows.

Let the dataset be m d-dimensional data $X=\left\{x_{1},x_{2},\cdots ,x_{m}\right\}$.

Step 1: Calculate the population mean *u* of the sample by:

(4) $$u=\frac{1}{m}\overset{m}{\underset{j=1}{\sum }}x_{j}$$

Step 2: Calculate the covariance matrix *S* of the sample by:

(5) $$S=\frac{1}{m-1}\overset{m}{\underset{j=1}{\sum }}\left(x_{j}-u\right){\left(x_{j}-u\right)}^{T}$$

Step 3: Perform feature decomposition on the covariance matrix and select the largest features in *S* to form a new feature vector.

#### 2.3.3. Linear Discriminant Analysis (LDA)

The basic idea of LDA is to project high-dimensional samples into the optimal discriminant vector space to achieve the purpose of extracting classification information and compressing the dimensions of the feature space [17]. The LDA method can find the optimal projection direction of the feature vector, which can maximize the ratio of the inter-class matrix to the intra-class dispersion matrix of the post-projection vector, thereby improving the recognition accuracy. The LDA feature vector was extracted using the following steps.

Step 1: Calculate the inter-class dispersion *S_b_* of the dataset *x* in the d-dimensional feature space by:

(6) $$S_{b}=\overset{L}{\underset{i=1}{\sum }}\left(u_{i}-u\right){\left(u_{i}-u\right)}^{T}$$

where $u_{i}$ is the mean vector of the $i^{\mathrm{th}}$ sample category, and *L* is the number of categories.

Step 2: Calculate the intra-class dispersion $S_{w}$ of the d-dimensional feature space by:

(7) $$S_{w}=\overset{L}{\underset{i=1}{\sum }}\overset{L}{\underset{k=1}{\sum }}\left(u_{i}-x_{k}\right){\left(u_{i}-x_{k}\right)}^{T}$$

Step 3: Solve the features of the matrix $S_{w}^{-b}S_{b}$ and select the vector consisting of the largest features as the new feature vector.

### 2.4. KNN-SVM Classifier

The K-nearest neighbor (KNN) algorithm is a popular machine learning algorithm. The principle of this algorithm is that if the majority of the k samples closest to a sample in a feature space belong to a specific category, then the sample also belongs to this category [31,32]. The distance can adopt the Euclidean distance, Manhattan distance, norm, etc. This study uses Euclidean distance, as given by:

(8) $$L\left(x_{i},x_{j}\right)={\left(\overset{n}{\underset{l=1}{\sum }}{\left|x_{i}^{\left(l\right)}-x_{j}^{\left(l\right)}\right|}^{2}\right)}^{\frac{1}{2}}$$

SVM [33] focuses on how to build a learning machine and how to implement classification on the basis of statistics and optimization. The idea of SVM is to find the best separation hyperplane in the feature space so that the intervals of the positive and negative samples on the training set are the largest, thus minimizing the structural risks. SVM is mainly used to research the classification of two categories. The commonly used methods are the one-to-one method and the one-to-many method for multi-classification problems [34]. The one-to-many method is the earliest and currently the most extensively used. This study adopts the one-to-many approach.

The SVM solves support vectors using quadratic programming, where storage and computation consume substantial machine memory and runtime. Therefore, SVM algorithms are challenging to use for processing large-scale training samples. Because of this problem, this study proposes a KNN-SVM classifier. The KNN-SVM classifier was constructed as follows:

For the classification of the samples to be tested, first, the KNN algorithm was used to select K values from the training samples that were the nearest neighbors of the samples to be tested. If K values belong to the same category, the samples to be tested belong to this category. If K values do not belong to the same category, K sample values are taken as the new training set, and the SVM training model is used. The kernel function of the SVM adopts the Gaussian radial kernel function, expressed by:

(9) $$k(x,x_{i})=exp(-\frac{\|x-x_{i}\|{}^{2}}{\sigma^{2}})$$

**Figure 13:**
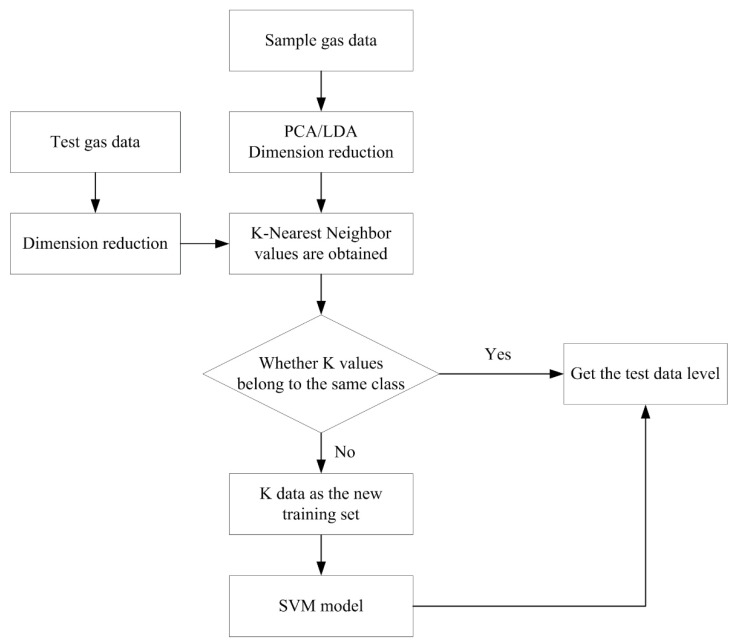
The process of the KNN-SVM algorithm.

The optimal values of the *k* in the KNN, the kernel width of the kernel function in the SVM, and the error multiplication parameter C were selected by the cross-validation of the sample. The classification model trained by the new training set had a better classification effect on the samples to be tested compared with the model trained by the original large-scale data. The process of the KNN-SVM algorithm is shown in Figure 13. Experimental results showed that compared with the traditional SVM algorithm, KNN-SVM was improved in both classification accuracy and speed, and it is suitable for large-scale data.

### 2.5. Classic Classifiers

Commonly used classification algorithms include the KNN algorithm, SVM algorithm, Decision Tree, Random Forest, etc. Decision Tree analysis is a classification algorithm for data mining. Decision Tree exists logically in the form of a tree, including root nodes, internal nodes, and leaf nodes. The root nodes contain a set of all data in the dataset. Each internal node is a judgment condition and includes a set of data in the dataset that satisfies all the conditions from the root node to the node. The data corresponding to the internal node are classified into two or more child nodes according to the testing result of the judgment condition of the internal node. The leaf node is the final category, and the data contained in the leaf node belong to the category [35]. Random Forest is constructed on the basis of multiple Decision Trees. The integrated learning idea of Bagging is adopted in a Decision Tree, and random feature selection is adopted in the process of training a Decision Tree. The steps for implementing a Decision Tree are as follows. Firstly, N samples are randomly selected from the original dataset, and then the training algorithm is performed on the N samples to obtain N decision trees. When using Random Forest for classification, the N Decision Trees are used for prediction, and the final classification is determined by voting for the final prediction result of each Decision Tree [36]. In the experiment, the above classic classifiers were compared with the proposed KNN-SVM classifier.

## 3. Results and Discussion

### 3.1. Simulation Results of the Flow Fields in Two Nasal Cavity Structures

**Figure 14:**
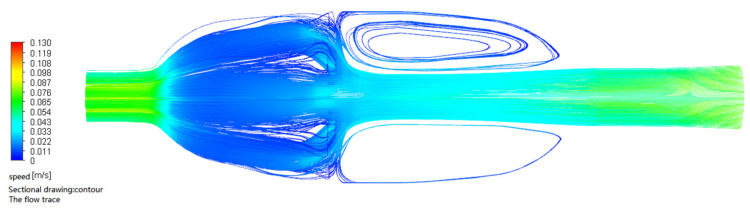
The flow field simulation cavity structure without steady flow plates.

**Figure 15:**
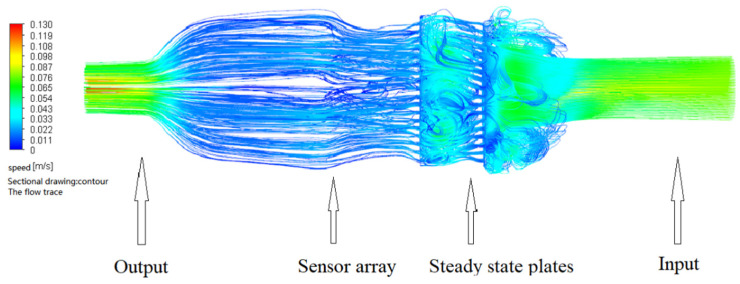
The flow field simulation cavity structure with steady flow plates.

The flow field simulations of the two nasal cavity structures are shown in Figure 14 and Figure 15. In the simulation, the inlet velocity was set at 0.1 m/s. It can be seen from the figures that for the device without steady flow plates, the gas inside the device returned after hitting the pipe wall, causing a disorder in the airflow around the sensor. Therefore, the measured gas data were unstable. By contrast, for the device with steady flow plates, the gas inside the device smoothly and evenly passed through the sensor array. As a result, there was a need to add steady flow plates in the nasal cavity structure.

**Figure 16:**
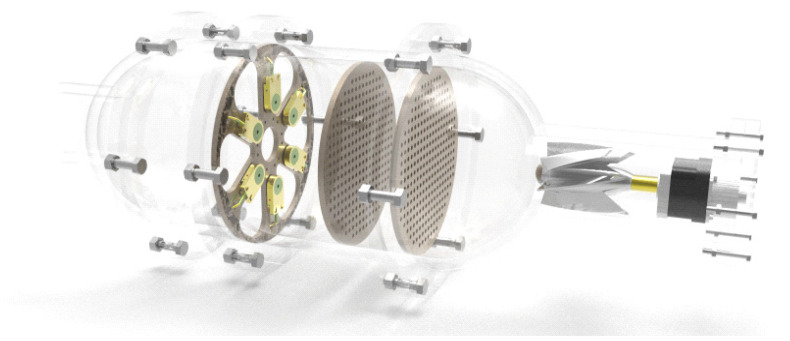
The overall design of the electronic nose.

The gas molecules must smoothly pass through the sensor array to make sure the odorous gas can be accurately detected by the gas sensor in the nasal cavity. It can be seen from the simulation results that the second nasal cavity structure was superior to the first one. Therefore, the second structure was selected to verify the feasibility of the designed e-nose. The overall design is shown in Figure 16.

### 3.2. Results of Apple Quality Classification

#### 3.2.1. Results of PCA and LDA

**Figure 17:**
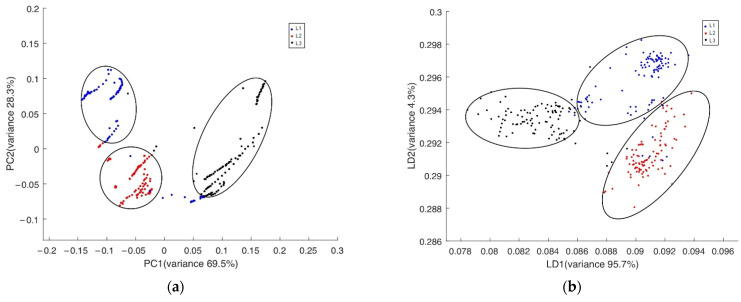
The grading results analyzed by PCA and LDA. (**a**) PCA, (**b**) LDA.

PCA and LDA were performed on the 300 sets of 18-dimensional data, and the partial features, variance contribution rates, and cumulative variance contribution rates corresponding to PCA and LDA were obtained, as shown in Table 4. For the LDA, since the contribution rate of the component from the third item was much lower than 1, it is not listed in the table. It can be seen in Table 4 that the variance contribution rate of the second component after PCA processing was not more than 1, the cumulative contribution rate of PC1 and PC2 was 97.8%, and the cumulative contribution rate of LD1 and LD2 by LDA was 99.96%. Thus, the cumulative contribution rates were all greater than 95%, indicating that the first two components of the two analytical methods could reflect all the information of the collected data. The grading results by PCA and LDA are shown in Figure 17. It can be seen from the figure that more than 90 percent of the data have no overlap, and the same kind of data are clustered together, which indicates that the designed e-nose system could accurately measure the odor data of one category of apples. In the data analysis section. we used Python 3.7 language and Pycharm 2020 (JetBrains, CZE).

#### 3.2.2. Rapid Classification of Apple

In this study, the dataset was divided with a ratio of 4:1; 240 sets of independent sample data were used as the training set to train the model in the test, and 60 sets of independent sample data were used as the test set. The PCA 2D feature vector, the LDA 2D feature vector, and the original 18-dimensional feature vector were input into the KNN-SVM classifier. Since LDA belongs to supervised dimensionality reduction, the projection vector obtained from the training set was used in the dimensionality reduction of the test set, and the data were input into the classifier after dimensionality reduction. The results are shown in Table 5. The recognition rate in the table takes an average of the recognition results in the test set. It can be seen that after the dimensions of the data were effectively reduced, the recognition accuracy was improved, and the training time was significantly reduced. Because of the cross-sensitivity of the gas sensors, the features of the original data affect each other. The generation of 2D linear independent feature vector groups using PCA and LDA not only reduces the data training time but also improves recognition accuracy.

The KNN algorithm was used as the training sample, and the model parameters of the SVM were constantly adjusted to learn the optimal classification hyperplane, which is the advantage of this algorithm. The proposed algorithm was compared with four popular classifiers: KNN, SVM, Decision Tree, and Random Forest. The data whose dimensions were reduced by PCA and LDA were used as the input, and the result with the highest accuracy was taken as the final result. The results are shown in Table 6. The data in the table expresses that the proposed classification method has certain advantages in accuracy and training time compared with other classification methods. The recognition accuracies of the four classic classifiers were 93.3%, 83%, 93%, and 91%, respectively. By contrast, the recognition accuracy of the proposed method was 97.78%. The training times of the four classic classifiers were 0.307 s, 0.858 s, 0.860 s, and 0.350 s, respectively, while that of the proposed method was 0.614 s. Overall, the training time of the proposed method was slightly longer than that of the traditional KNN and Decision Tree, whereas the recognition accuracy of the former was significantly improved. The e-nose device used in this study had a higher recognition rate for determining apple quality than the device used by Nturambirwe [33]. Compared with the studies of Rasekh et al. [37] and Tatli et al. [38], the e-nose technology applied in apple detection in this study achieved higher accuracy.

## 4. Conclusions

In this study, an e-nose system with an optimized nasal cavity structure was designed. The flow fields of the two nasal cavity structures with and without the steady flow plates were simulated. It was found that the airflow of the nasal cavity with the constant flow plates was more even and stable. Therefore, the steady flow plate was adopted in the e-nose to grade the apples on the basis of their quality. PCA and LDA were performed for the data collected from the designed e-nose. The features of the 18-dimensional gas data were extracted. Although some data were overlapped, they could still be generally distinguished. Then, the KNN-SVM classifier was constructed, and the original 18-dimensional data, dimension-reduced PCA data, and dimension-reduced LDA data were used as the input of the classifier. The accuracy of classification for the LDA data was 97.78%. On the basis of a comparison between the proposed classifier and other popular classifiers, it was discovered that the former had a better performance in both accuracy and speed. The e-nose device only took several seconds for data processing after the pre-work of detection was ready, which demonstrates the effectiveness of the device for apple grading. The method might be applicable to other fruit quality assessments.

## Figures and Tables

**Table 1:** Descriptions of the three levels of apple quality: L1, L2, and L3.

Level	External Quality	Internal Quality
L1	No stab wounds, broken skin, crushed wounds, disease wounds, insect wounds, rot, or shrinkage on the surface of apples; smooth and rosy surface.	No rot, shrinkage, or dryness inside the apples, which can be eaten normally.
L2	Slight skin damage, stab wounds, or frostbite appear on the surface of the apple, and there are a few black spots.	No rot, shrinkage, or dryness inside apples.
L3	The surface of the apple is obviously damaged, with pests and disease, and there is decay or shrinkage.	Rot, shrinkage, or dryness inside the apples, which cannot be eaten normally.

**Table 2:** Details of sensor.

Sensor	Target Gas	Manufacturer
MQ-9	CO, CH_4_	Hanwei Electronics Co., Ltd., Zhengzhou, China
MQ-3	alcohols	Hanwei Electronics Co., Ltd., Zhengzhou, China
MQ-6	C_3_H_8_, C_4_H_10_	Hanwei Electronics Co., Ltd., Zhengzhou, China
MQ-8	H_2_	Hanwei Electronics Co., Ltd., Zhengzhou, China
MQ-2	liquefied gas	Hanwei Electronics Co., Ltd., Zhengzhou, China
MQ-135	NH_3_, benzene vapors	Hanwei Electronics Co., Ltd., Zhengzhou, China

**Table 3:** The boundary conditions for simulating the flow field.

Parameters	Values
Simulated state	Steady state
model	Laminar
Air density (kg/m^3^)	1.225
Dynamic Viscosity (Pa·s)	1.83 × 10^−5^
Inlet	Velocity—inlet
Outlet	Pressure—outlet

**Table 4:** The features and component contribution rate.

	Component	Feature	Variance Contribution Rate (%)	Cumulative Contribution Rate (%)
PCA	PC1	1.048	69.52	69.52
	PC2	0.427	28.34	97.87
	PC3	0.015	1.00	98.87
	PC4	0.012	0.83	99.7
LDA	LD1	3.046	95.68	95.68
	LD2	0.137	4.28	99.96

**Table 5:** The influence of the feature vector with the result of training.

Feature Vector	Training Time (Unit: t/s)	Recognition Rate (%)			
		L1	L2	L3	Average Recognition Rate
The original 18-dimensional feature vector	3.653	93.35	92.45	94.19	93.33
PCA 2D feature vector	0.618	96.66	95.45	97.24	96.45
LDA 2D feature vector	0.614	98.50	96.66	98.18	97.78

**Table 6:** The comparison results of the five kinds of classifiers.

Method	Training Time (Unit: s)	Recognition Rate (%)
KNN	0.307	93.30
SVM	0.858	83.00
Random Forest	0.86	93.00
Decision Tree	0.35	91.00
The proposed algorithm of this study	0.614	97.78

## Data Availability

Not applicable.
